# Establishment of a real-time fluorescent quantitative PCR detection method and phylogenetic analysis of BoAHV-1

**DOI:** 10.1186/s12917-024-04025-8

**Published:** 2024-05-07

**Authors:** Lihui Xu, Guiyang Ge, Dongli Li, Jianming Li, Qinglong Gong, Kun Shi, Fei Liu, Naichao Diao, Zhenzhen Cui, Yingyu Liu, Xue Leng, Rui Du

**Affiliations:** 1 College of Chinese Medicine Materials, Jilin Agricultural University, Changchun, 130118 Jilin China; 2 College of Animal Science and Technology, Jilin Agricultural University, Changchun, 130118 Jilin China

**Keywords:** BoAHV-1, RT‒qPCR, Phylogenic analysis

## Abstract

**Background:**

*Infectious bovine rhinotracheitis* (IBR), caused by *Bovine alphaherpesvirus-1* (BoAHV-1), is an acute, highly contagious disease primarily characterized by respiratory tract lesions in infected cattle. Due to its severe pathological damage and extensive transmission, it results in significant economic losses in the cattle industry. Accurate detection of BoAHV-1 is of paramount importance. In this study, we developed a real-time fluorescent quantitative PCR detection method for detecting BoAHV-1 infections. Utilizing this method, we tested clinical samples and successfully identified and isolated a strain of BoAHV-1.1 from positive samples. Subsequently, we conducted a genetic evolution analysis on the isolate strain’s gC, TK, gG, gD, and gE genes.

**Results:**

The study developed a real-time quantitative PCR detection method using SYBR Green II, achieving a detection limit of 7.8 × 10^1^ DNA copies/μL. Specificity and repeatability analyses demonstrated no cross-reactivity with other related pathogens, highlighting excellent repeatability. Using this method, 15 out of 86 clinical nasal swab samples from cattle were found to be positive (17.44%), which was higher than the results obtained from conventional PCR detection (13.95%, 12/86). The homology analysis and phylogenetic tree analysis of the gC, TK, gG, gD, and gE genes of the isolated strain indicate that the JL5 strain shares high homology with the BoAHV-1.1 reference strains. Amino acid sequence analysis revealed that gC, gE, and gG each had two amino acid mutations, while the TK gene had one synonymous mutation and one H to Y mutation, with no amino acid mutations observed in the gD gene. Phylogenetic tree analysis indicated that the JL5 strain belongs to the BoAHV-1.1 genotype and is closely related to American strains such as C33, C14, and C28.

**Conclusions:**

The established real-time fluorescent quantitative PCR detection method exhibits good repeatability, specificity, and sensitivity. Furthermore, genetic evolution analysis of the isolated BoAHV-1 JL-5 strain indicates that it belongs to the BoAHV-1.1 subtype. These findings provide a foundation and data for the detection, prevention, and control Infectious Bovine Rhinotracheitis.

**Supplementary Information:**

The online version contains supplementary material available at 10.1186/s12917-024-04025-8.

## Introduction

BoAHV-1 is a significant pathogen in the global livestock industry that is capable of causing upper respiratory tract diseases in cattle, such as rhinitis and bronchitis. Additionally, BoAHV-1 can lead to other reproductive complications, including abortions, fetal deformities, and reproductive tract infections in cows [1]. Infected cattle initially exhibit clinical infections and parallelly establish latent infections, which later transform into persistent infections, with intermittent shedding of the virus into the environment. In a livestock farm, if one dairy cow becomes infected with BoAHV-1, the virus can spread rapidly throughout the entire cattle herd within a relatively short period, establishing a lifelong latent period [2]. During periods of immunosuppression, BoAHV-1 can undergo reactivation. This reactivation can be induced by corticosteroids or stress, leading to the activation of latent viruses. Subsequently, the virus can spread through respiratory secretions, ocular secretions, reproductive tract secretions, and even through infected bull semen transmission [3, 4]. BoAHV-1 infection can also suppress the host's immune system, resulting in secondary bacterial infections that further worsen the condition [5]. In addition, BoAHV-1, along with other respiratory viruses, such as bovine viral diarrhea virus (BVDV), bovine respiratory syncytial virus (BRSV), bovine parainfluenza virus type 3 (BPIV-3), and bacterial coinfections, can cause bovine respiratory disease complex (BRDC), inflicting significant damage to the global cattle industry [6].

The disease first appeared in the 1950s in Colorado, USA, and subsequently spread to Europe through trade and commerce [7]. In recent years, there have been reports of BoAHV-1 infections reemerging in some countries where it was not previously reported, as well as in countries that had previously declared BoAHV-1 eradicated [8]. A meta-analysis revealed that the overall infection rate of BoAHV-1 in Chinese cattle is 40%, with the Inner Mongolia region showing a particularly high rate of 66%. These findings suggest that BoAHV-1 is widely prevalent in cattle populations in China and has shown an increasing trend in recent years [9]. As of the end of 2022, the total cattle inventory in China reached 102.16 million head, showing an increase of 3.99 million head compared to the previous year-end, representing a growth rate of 4.1%. The large population of cattle and their rapid growth rate have led to the accelerated spread of BoAHV-1, which has hindered the development of China's cattle industry. Currently, cell culture, polymerase chain reaction (PCR), real-time fluorescence quantitative PCR, enzyme-linked immunosorbent assay (ELISA), and virus neutralization assay have been developed to detect BoAHV-1. Among them, Real-Time Fluorescent Quantitative PCR is widely used because of its high sensitivity, accuracy and specificity compared with other methods [10]. Therefore, in this study, a Real-Time Fluorescent Quantitative PCR method was developed to support the early prevention and transmission of BoAHV-1.

The evolutionary dynamics of numerous viruses within the Alphaherpesvirinae subfamily have been partially attributed to the process of recombination [11]. Recombination plays a particularly significant role in the evolution and diversity of Alphaherpesviruses. However, there is currently limited research regarding genetic variation in BoAHV-1 [12]. To promptly understand the genetic evolution characteristics and epidemiological trends of BoHV-1 and formulate more effective prevention and control strategies, it is imperative to investigate this aspect further. Therefore, in this study, a Real-Time Fluorescent Quantitative PCR method based on SYBR Green II was developed to support the detection of BoAHV-1.The genetic relationships between BoAHV-1 strain JL5 and other isolates were analyzed to provide insights for the development of regional prevalent strain vaccines against BoHV-1. This endeavor aims to contribute scientific evidence and support for the prevention and control of BoAHV-1.

## Results

### Establishment of the standard curve for qPCR

**Fig. 1:**
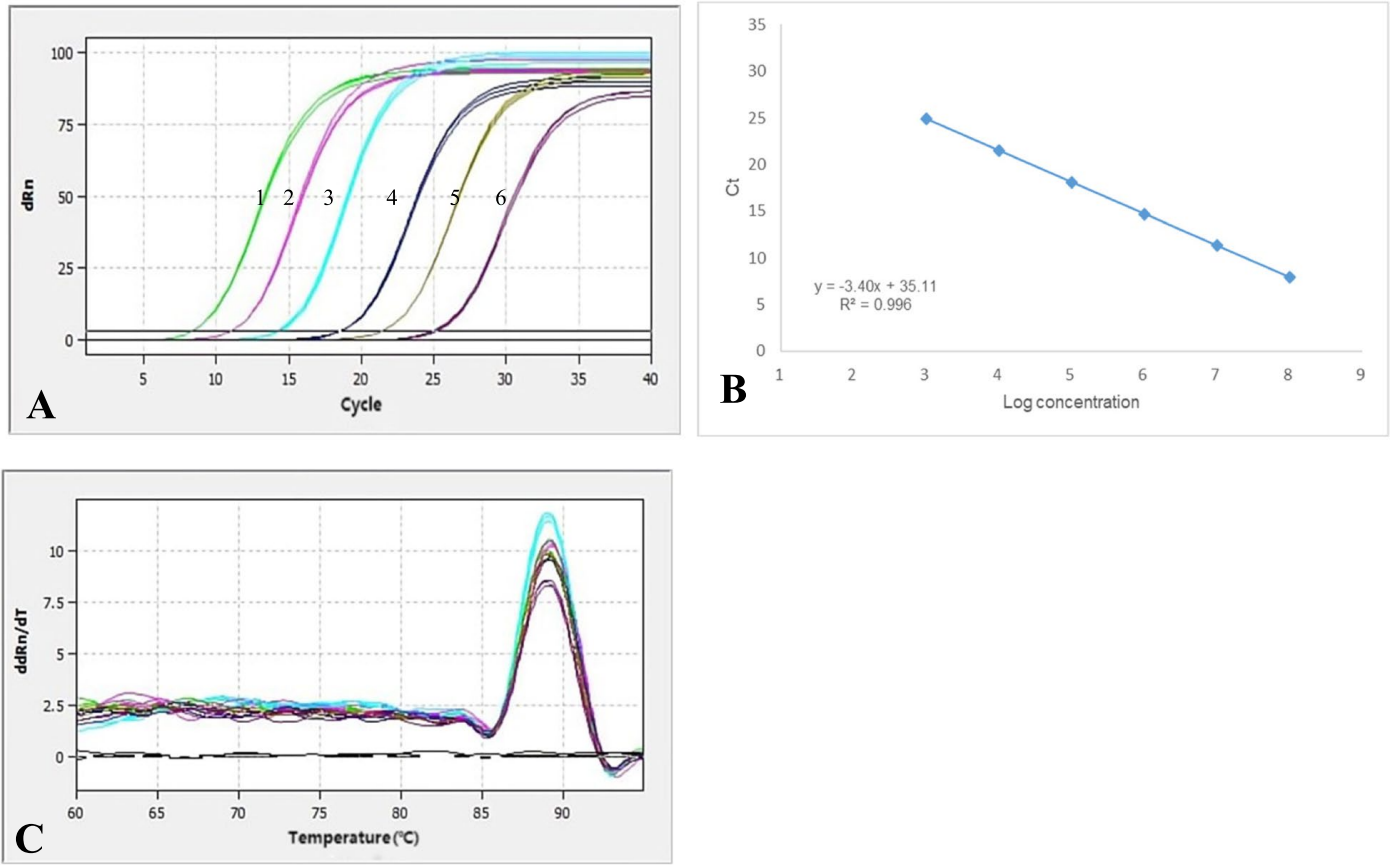
An amplification plot, melting curve analysis, and a standard curve. **A** A plot of amplification: the Y-axis represents the fluorescence intensity, and 1–6 show plasmid concentrations, ranging from 7.80 × 10^3^ ~ 7.80 × 10^8^ copies/μl. **B** Standard Curve: The X-axis represents the copy number, which ranges from 7.80 × 10^3^ ~ 7.80 × 10^8^ copies/μl. The corresponding Ct values are represented on the Y-axis. **C** The melting curve with a melting peak at 89.08 °C ± 0.5 °C. The peak is single and of good quality

The results showed that the amplification curves of the TK gene had good reproducibility, and the fluorescence intensity varied regularly with the plasmid concentration (Fig. 1A). The linear regression equation of the standard curve for the TK gene was y = -3.40X + 35.11. The correlation coefficient R^2^ was 0.996, and the amplification efficiency (E) was 96.83% (Fig. 1B). The melting curve analysis showed that all positive samples exhibited a single peak with a melting temperature of 89.08 ± 0.5 °C (Fig. 1C). Meanwhile, no melting curves or dimer curves were observed in the negative control.

### Specificity, sensitivity and reproducibility of RT‒qPCR detection

**Fig. 2:**
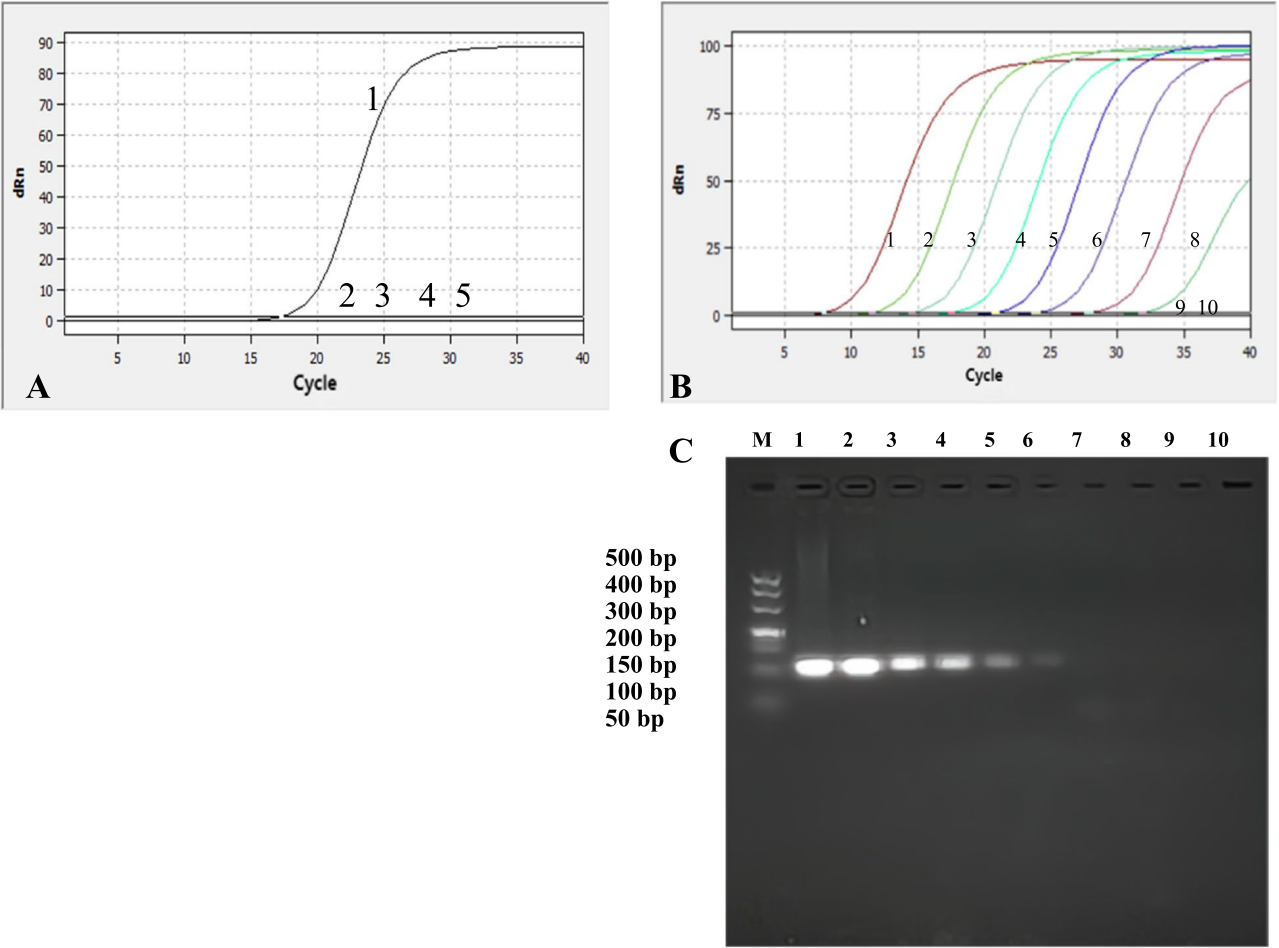
Specificity and sensitivity test curve of real-time PCR of TK. **A** Specificity analysis; 1–5: BoAHV-1, BVDV, BRSV, BPIV-3 and ddH2O; **B** TK 1–9: 7.80 × 10^8^, 7.80 × 10^7^, 7.80 × 10^6^, 7.80 × 10^5^, 7.80 × 10^4^, 7.80 × 10^3^, 7.80 × 10^2^, 7.80 × 10^1^, 7.80 × 10^0^copies/µL; 10: Negative control; **C** Sensitivity test of conventional PCR of TK; Lane M: DL500 DNA Marker; Lane M1-9: 7.80 × 10^8^, 7.80 × 10^7^, 7.80 × 10^6^, 7.80 × 10^5^, 7.80 × 10^4^, 7.80 × 10^3^, 7.80 × 10^2^, 7.80 × 10^1^, 7.80 × 10^0^copies/µL; 10: Negative

The BoAHV-1 strain exhibited strong fluorescence signals through specificity analysis, while no signals were detected in BVDV, BRSV and BPIV-3, indicating excellent specificity of the established detection method (Fig. 2A). And the twenty BoAHV-1 negative sampls were all negative by this method. Based on the analysis of the amplification curves and standard curves, the LoDs of TK were detected by real-time qPCR to be 7.80 × 10^1^ copies/µL (Fig. 2A, B). Conventional PCR results showed that the detection limit of the recombinant positive plasmid pMD18-T-TK was 7.80 × 10^3^ copies/µL (Fig. 2C). Therefore, real-time qPCR was 100 times more sensitive than conventional PCR. The twenty BoAHV-1 positive sampls were all positive by this method. The results of the reproducibility test showed that the intrabatch coefficients of variation ranged from 0.10% to 0.71%, and the interbatch coefficients of variation ranged from 0.03% to 3.39%. These results indicate that the real-time fluorescence quantitative PCR assay is highly reproducible.

### Detection of the clinical samples

The detection of 86 clinical samples of nasal swabs showed a positive rate of 13.95% (12/86) by conventional PCR. The positive detection rate of the TK gene using SYBR Green II real-time PCR was 17.44% (15/86), and the concordance rate with the positive detection rate of conventional PCR was 80% (12/15) (Table 1). The results indicate that the SYBR Green II real-time PCR assays established in this study are more sensitive than conventional PCR.

**Table 1:** Results of clinical sample testing

Detection Method	Number of Positives/Total Number	Positivity Rate
conventional PCR	12/86	13.95%
Quantitative PCR(TK)	15/86	17.44%

### Virus isolation and identification

**Fig. 3:**
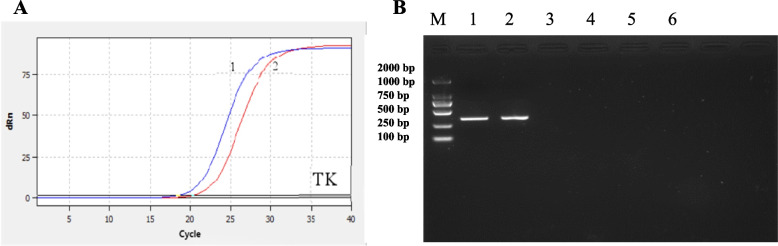
Identification of the JL5 strain of the virus. **A** Fluorescent quantitative PCR identification results: 1–3: BoAHV-1 positive control, isolated BoAHV-1 strain detection, negative control. **B** Conventional PCR identification results: Lane M: DL2000 DNA marker; Lane 1: BoAHV-1 standard positive strain; Lane 2: Isolated BoAHV-1 strain; Lane 3: Negative control of BoAHV-1 (MDBK cells); Lane 4: BVDV; Lane 5: BRSV; Lane 6: BPIV-3

The nasal swab fluid identified as positive by qPCR was inoculated onto MDBK cells. Cellular pathology was observed in the first passage post-inoculation, manifested by phenomena such as round shrinkage, network formation, and aggregation resembling grape-like clusters. Approximately 80% of the cells exhibited pathological changes by 40 h post-inoculation, with cells gradually detaching and undergoing apoptosis over prolonged incubation periods. Similar observations were noted in the positive control group, while cells in the blank control group maintained normal morphology. Subsequently, after purification and cultivation, a viral isolate with a titer of 1 × 10^^7.5^ TCID_50_/mL was obtained. The application of the previously established real-time PCR for detection showed amplification signals for the isolated virus (Fig. 3A), while no target bands were obtained for the BVDV, BRSV and BPIV-3 genes (Fig. 3B). In conventional PCR testing, positive bands were observed in the positive control group and the isolated virus strain group, while no bands were detected in the other groups.

### Phylogenetic analysis

The fragment lengths of gE, TK, gG, gD and gC were 1728 bp, 1080 bp, 1335 bp, 1254 bp and 1527 bp, respectively, by sequencing. In the gC gene, nucleotide homology with BoAHV-1 type reference strains ranges from 98.2% to 99.9%. The amino acid sequence analysis revealed a nonsynonymous mutation at position 228 (A to T) and a mutation at position 504 (A to D). In the gD gene, nucleotide homology with BoAHV-1 type reference strains ranges from 98.5% to 100.0%. Amino acid sequence analysis showed no amino acid mutations, indicating that the gD gene is relatively conserved in genetic evolution. Concerning the gE gene, nucleotide homology with BoAHV-1 type reference strains is between 99.4% and 100.0%. Amino acid sequence analysis identified nonsynonymous mutations at positions 62 (D to G) and 86 (L to H). In the gG gene, nucleotide homology with BoAHV-1 type reference strains is in the range of 98.9% to 99.9%. Amino acid sequence analysis revealed nonsynonymous mutations at positions 4 (A to T) and 341 (N to I). In the TK gene, nucleotide homology with BoAHV-1 type reference strains is between 99.4% and 99.8%. Amino acid sequence analysis found synonymous mutations at position 239 and a nonsynonymous mutation at position 85 (H to Y) (Table 2).

**Table 2:** Nucleotide and amino acid changes in gC, gE, gG, and TK genes

gene name	nucleotide sites	nucleotide variation	amino acid site	amino acid variation
gC	682	G → A	228	A → T
	1511	C → A	504	A → D
gE	185	A → G	62	D → G
	257	T → A	86	L → H
gG	10	G → A	4	A → T
	1022	A → T	341	N → I
TK	253	C → T	85	H → Y
	717	T → C	239	N → N

The nucleotide and amino acid alignment sequences are of the BoAHV-1.1 type strain, as shown in Table 2

**Fig. 4:**
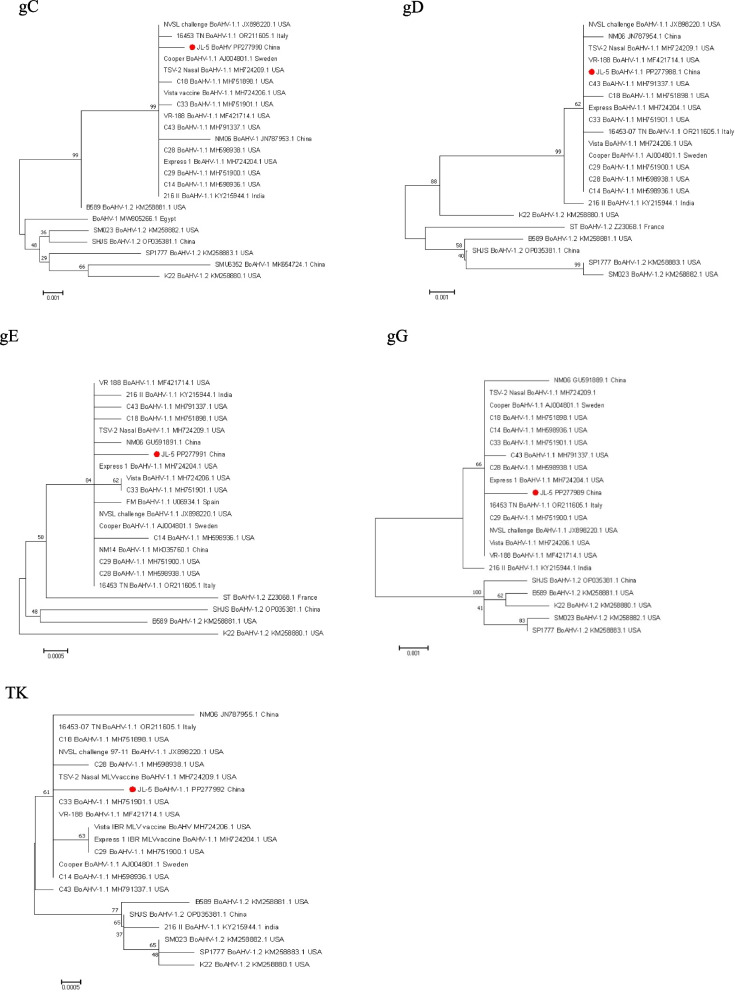
**A-E** Phylogenetic tree analysis of gC, gD, gE, gG and TK genes

In summary, our analysis revealed a high homology between the JL5 strain and BoAHV-1.1 subtype strains, especially with U.S. strains such as C33, C14, C43, C28, and vaccine strains. The homology with strains from other countries, including China's NM06 strain, was relatively low. Therefore, we speculate that the BoAHV-1 JL5 strain may originate from the U.S. strains C33 and C14, possibly introduced through international trade. U.S. vaccine strains MH724206.1 and MH724209.1 showed high homology with U.S. strains C14, C18, C29, and C33, suggesting that U.S. BoAHV-1 vaccines might be effective in preventing and controlling the JL5 strain. In conclusion, genetic evolutionary tree analysis based on five genes indicates that the JL5 strain belongs to the BoAHV-1.1 subtype rather than the BoAHV-1.2 subtype (Fig. 4).

Each strain is listed by GenBank accession number, geographic origin, and collection date. Bootstrap values are shown as percentages at each tree node. Scale bar indicates substitutions per site.

## Discussion

Bovine herpesvirus 1 (BoAHV-1) is recognized as a significant pathogen causing bovine respiratory disease complex, contributing to substantial economic losses in the global cattle farming industry. The rapid development of China's beef and dairy products sector in recent years has resulted in an increase in cattle transportation frequency. Concurrently, the importation of dairy cows and expansion of cattle herds have contributed to the rising prevalence of BoAHV-1. Although BoAHV-1 vaccines are available to mitigate clinical symptoms, their effectiveness in controlling latent infections remains suboptimal [13, 14]. Research has shown that sheep and goats can be infected with BoAHV-1 and develop diseases. BoAHV-1 antibodies have been detected in captive Asian elephants [15], and the virus can also be isolated from healthy individuals of antelope, zebras, ferrets, and minks [16]. Recently, it was discovered that BoAHV-1 is also present in dromedary camels [17]. This indicates that the range of BoAHV-1 infection is continuously expanding and may have an impact on different animal populations. Consequently, the complete eradication of BoAHV-1 has become exceedingly challenging, emphasizing the urgency for the development of a rapid and reliable detection method.

Currently, there are various methods available for detecting infectious bovine rhinotracheitis virus (BoAHV-1), and among them, the most widely used and technically mature approaches include virus isolation, virus neutralization tests, enzyme-linked immunosorbent assay (ELISA), and polymerase chain reaction (PCR) [18–21]. Among these methods, fluorescence quantitative PCR (qPCR) is the most widely applied technique for laboratory detection. It is also the BoAHV-1 detection method recommended by the World Organization for Animal Health (WOAH). It does not require gel electrophoresis to obtain results, thus compensating for the time-consuming aspect of conventional PCR and making it suitable for testing a large number of clinical samples. Compared to TaqMan probe-based real-time qPCR, SYBR Green II-based real-time qPCR does not require the design of specific probes. This simplifies the experimental procedure and reduces the overall cost, making it advantageous for widespread promotion and application [22]. Certainly, real-time qPCR also has certain limitations. For instance, it may require higher personnel and equipment demands compared to some other methods. It cannot detect infections caused by vaccines with deleted TK genes, as the results of this method would be negative. Additionally, there would be better applications in the future if the detection costs and requirements could be reduced.

The selection of target genes for fluorescence quantitative PCR (qPCR) is of paramount importance in BoAHV-1 detection. The genome of BoAHV-1 is extensive and encompasses numerous essential genes, including gB, gC, gD, gE and TK [23–27]. In this study, we chose the TK gene as target genes because the TK gene is a key and conserved gene responsible for maintaining continuous BoAHV-1 infection, and it is also one of the major virulence genes, making it a good candidate target for BoAHV-1 detection. Moreover, as the TK gene is a nonessential gene, it is also one of the main target genes for developing BoAHV gene-deleted vaccines [28]. This also implies that our developed detection method can distinguish between natural BoAHV-1 infection in cattle and infection caused by BoAHV-1 gene-deleted vaccines targeting the TK gene. The utilization of these key genes as targets in our research contributes to the advancement of BoAHV-1 detection methods and aids in the effective control and management of BoAHV-1-related outbreaks. BoAHV-1 is a double-stranded DNA virus belonging to the *Varicellovirus* genus in the *Alphaherpesvirinae* subfamily. It has a genome size of approximately 140 kb [24]. In BoAHV-1, the TK gene is a nonessential gene located in the UL region of BoAHV-1. This gene primarily encodes BoAHV-1 thymidine kinase, which is an essential enzyme in the thymidine synthesis pathway. TK serves as one of the major virulence genes in herpesviruses and is also a nonessential gene in herpesvirus replication, playing a significant role in nucleic acid metabolism [29]. When the TK gene is deleted, it significantly reduces the virulence and replicative capacity of BoAHV-1. Therefore, in the development of BoAHV-1 gene-deletion vaccines, the TK gene is often targeted for knockout [28]. Furthermore, the gene sequence of BoAHV-1 TK is relatively conserved, making it a promising candidate target for BoAHV-1 detection.

Therefore, this study successfully established the SYBR Green II RT‒qPCR detection method for BoAHV-1 targeting the TK gene. The method exhibited a good linear relationship of the standard curves, meeting the needed criteria, with correlation coefficients (R^2^) and amplification efficiencies (E) within acceptable ranges. When using the established detection method on clinical samples, we observed a positivity rate of 17.44% (15/86). This indicates a relatively high prevalence of BoAHV-1 clinical infections. The detection method we have developed has a detection limit of 7.8 × 10^^1^ copies/μL. Compared to the fluorescence quantitative PCR method established by Wang et al. [30] (5.747 × 10^^2^ copies/μL), it demonstrates higher sensitivity. In contrast to the conventional duplex PCR methods established by Xu et al. [31] with detection limits of 2.3 × 10^^3^ copies/μL and 2.4 × 10^^3^ copies/μL respectively, our method not only offers higher sensitivity but also significantly saves experimental time, thereby enhancing the efficiency of clinical sample detection. Moreover, compared to the LAMP method established by Gao et al. l [32] (with a detection limit of 1 × 10^^3^ copies/μL and a duration of 1 h), our method exhibits a similar experimental duration but with higher sensitivity.Since we were unable to obtain isolates of Bubaline alphaherpesvirus 1 (BuAHV-1) and Bovine alphaherpesvirus 5 (BoAHV-5), to ensure specificity in our results, we performed sequence alignment analysis of the TK gene sequences of BuAHV-1 (KU936049.1) and BoAHV-5 (NC_005261.3) during primer design. We synthesized specific primers to ensure the specificity of our detection method. Additionally, we utilized the specific primers for BuAHV-1 and BoAHV-5 as outlined in the study by Peletto et al. [33] to detect clinical samples. The results indicated that both BuAHV-1 and BoAHV-5 were negative.Additionally, we tested for cross-reactivity with Bovine Viral Diarrhea Virus (BVDV), Bovine Respiratory Syncytial Virus (BRSV), and Bovine Parainfluenza Virus-3 (BPIV-3), and the results showed no cross-reactivity. This indicates that our detection method is specific and suitable for detecting BoAHV-1 in complex clinical samples from cattle (Fig. 2A). The detection method provide an effective means for the early detection of BoAHV-1, reducing the risk of virus shedding from infected cattle and offering crucial technical support for the control and prevention of bovine infectious rhinotracheitis.

In this study, during the isolation of suspected viral samples, characteristic cytopathic effects (CPE) were observed after inoculating MDBK cells. The CPE showed a distinctive appearance, with cells forming grape-like clusters and becoming rounded, and as the culture time increased, the cells detached and appeared as individual cells. The established fluorescent quantitative detection method was utilized to confirm that the isolated strain was BoAHV-1. This strain was subsequently named BoAHV-1 JL5. The TCID_50_ of the virus strain was 1 × 10^7.5^ TCID_50_/mL, which is similar to the TCID_50_ of the Inner Mongolia BoAHV virus strain isolated by Zhang Pengfei [34]. Our further analysis of JL5, including homology and genetic evolution analysis, indicates that the BoAHV-1 JL5 strain belongs to the BoAHV-1.1 type (Fig. 4), consistent with the clinical observations we made during sample collection. The BoAHV-1.1 type primarily affects the respiratory tract, and infected cattle may exhibit symptoms such as coughing, tearing, and the discharge of purulent secretions from the nasal cavity and eyes [35]. The multiple sequence alignment results indicate that the gC, TK, gG, and gE genes of the BoAHV-1 JL5 strain have relatively few variations, and the gD gene shows no amino acid mutations. Additionally, the TK genes exhibit synonymous mutations (Table 2), which suggests that the JL5 strain shows good conservation. This is consistent with the results of the sequence analysis of the gC, gI and VP22 genes of the NM06 strain isolated in Inner Mongolia by Bai [36] and Hu et al. [37]. Combining the genetic variation analysis of the JL5 strain isolated in Jilin Province in this experiment, it can be concluded that BoAHV-1 strains isolated from the Jilin Province region are relatively conserved. Currently, there is limited information on the isolation, biological characteristics, and genetic evolution of BoAHV-1 epidemic strains in China. Therefore, the isolation of epidemic strains, analysis of key genes in isolated strains, and exploration of their biological characteristics are of significant importance in enriching our understanding of BoAHV-1 outbreak strains.

In conclusion, our analysis reveals a high homology between the JL5 strain and BoAHV-1.1 subtype strains, especially when compared to strains such as C33, C14, C43, and C28 and vaccine strains from the United States. In contrast, its homology is lower when compared to the Chinese NM06 strain and strains from other countries. Therefore, we hypothesize that the BoAHV-1 JL5 strain may have been introduced through international trade activities, possibly originating from the U.S. strains such as C33 and C14. Moreover, U.S. vaccine strains MH724206.1 and MH724209.1 exhibit significant homology with strains such as C14, C18, C29 and C33. Consequently, we speculate that U.S. BoAHV-1 vaccines may also be applicable in preventing and controlling the JL5 strain. In summary, the genetic evolutionary tree analysis of five genes clearly indicates that the JL5 strain belongs to the BoAHV -1.1 subtype rather than the BoAHV-1.2 subtype.

## Conclusion

This study successfully established a SYBR Green II real-time quantitative PCR detection method for BoAHV-1. The developed method demonstrates high sensitivity, good specificity, and rapid results, making it suitable for clinical diagnosis. Furthermore, this method was successfully applied to identify a new BoAHV-1 JL5 strain. Systematic phylogenetic analysis revealed that the gC, TK, gG, gD and gE genes of the BoAHV-1 JL5 strain clustered together with the reference strain of the BoAHV-1.1 type, indicating their close evolutionary relationship. This study provides new data for the epidemiological study of BoAHV-1 in China and lays the foundation for the development of novel BoAHV-1 detection technology and vaccines.

## Materials and methods

### Virus strains, clinical samples and primer design

BVDV, BRSV, and BPIV-3 were preserved at the Economic Animal Infectious Disease Laboratory of Jilin Agricultural University and were tested by the RT‒qPCR method [38]. Eighty-six cow nasal swabs were collected randomly from farms in Jilin province, some of the cows exhibit symptoms of increased secretion from the eyes and nose.

From the National Center for Biotechnology Information (NCBI) database (https://www.ncbi.nlm.nih.gov), the TK, gC, TK, gG, gD and gE genes of BoAHV-1 (GenBank accession number: AJ004801) were downloaded as reference sequences. The software Primer 5.0 was used to design one pair of primers suitable for fluorescence quantitative PCR in the conserved region of TK that were suitable for real-time fluorescent quantitative PCR. Additionally, primers for the identification of the full-length sequences of the gC, TK, gG, gD and gE (including gE1 and gE2) genes were also designed. The primer sequences are listed in Table 3. These primers were synthesized by Shanghai Sangon Biotech Co., Ltd. (Shanghai, China).

**Table 3:** Primer Sequence Information

primer	sequences(5'-3')	Length(bp)
qTK-F	GTGCCTCTGCTACCCCTTCG	114
qTK-R	CACACGACGAGGTTGGCC	
F	GGCTCTACCGCACGGGCACCTCT	362
R	GCGGCTCTCGTCTCGCAGCATTT	
gC-F	GGGGCCCCGCGCCTACAGG	1587
gC-R	CACGACCGCCGAGAGACCGCC	
TK-F	AGGCGCGCACGTCGGTCG	1128
TK-R	GCGAGCGCAGGGGCAGCTTTATA	
gG-F	CGCAAGCGCGAGCACACGAC	1389
gG-R	GGTCGGCCGTGGGG	
gD-F	TGCTGCGCAG0GGCGAAC	1302
gD-R	AGGGGGCGGGGGGAG	
gE1-F	CGAAAAGGGCATTTGGCAAT	1128
gE1-R	ACACGGTCTCGGAGCGGTAC	
gE2-F	CTCCGAGACCGTGTACAGCC	1028
gE2-R	GATGGACACGATGGAGCCTT	

### Standard plasmid construction

DNA of BoAHV-1 was extracted (OMEGA USA) and amplified by PCR with qTK-F and qTK-R, and the target fragments of the amplified fragments were ligated to the pMD18-T vector.

These recombinant plasmids were sent to Shanghai Sangon Biotech Co., Ltd. (Changchun, China) for synthesis and sequencing. The correctly sequenced plasmids were quantified using a nucleic acid analyser from Shimadzu Corporation (China). The sample copy number calculation formula was used to convert the copy number of the positive standard into appropriate units.

Formula: Sample copy number = [concentration (ng/µL) × Avogadro's constant (NA) × 10^–9^]/(660 × length of recombinant plasmid DNA in base pairs).

### Establishment of a standard curve for qPCR

Fluorescence quantitative PCR (qPCR) was performed using 6 different concentrations of recombinant positive plasmids, pMD18-T-TK (ranging from 10^3^ to 10^8^ copies/µL), as templates. A 25 µl amplification system was established with the following components: 12.5 µl of TB Green Premix Ex Taq II (Japan Takara), 9.5 µl of ddH_2_O, 1 µl of BoAHV-1-F (10 mM), 1 µl of BoAHV-1-R (10 mM), and 1 µl of the template. The qPCR was carried out in eight tubes by fluorescence quantitative PCR in a qTOWER3 G instrument (Analytik Jena AG, Jena, Germany). The PCR conditions consisted of an initial denaturation step at 95 °C for 30 s, followed by 40 cycles of denaturation at 95 °C for 5 s and annealing at 55 °C for 30 s. A negative water control was included. The amplification reactions generated cycle threshold (Ct) values, and the results were analysed to construct the standard curve. The standard curve was generated by monitoring the fluorescence signals of SYBR Green II during qPCR amplification.

### Specificity, sensitivity and reproducibility testing

To exclude any cross-reactivity between BoAHV-1 and other bovine viral pathogens, cDNA samples from BoAHV-1, BVDV, BRSV and BPIV-3 were used as templates in SYBR Green II real-time quantitative PCR. This approach allows for specific detection and quantification of each viral pathogen, ensuring the accuracy and reliability of the results. water was used as a blank control. Simultaneously, we employed the PCR method established in the laboratory to confirm 20 negative clinical samples for BoAHV-1 to test the specificity of this method. The primer sequences are listed in Table 3.

The sensitivity of BoAHV-1 Real-Time Fluorescent Quantitative PCR was determined by using a tenfold serial dilution of recombinant positive plasmid pMD18-T-TK (7.80 × 10^8^、7.80 × 10^7^、7.80 × 10^6^、7.80 × 10^5^、7.80 × 10^4^、7.80 × 10^3^、7.80 × 10^2^、7.80 × 10^1^、7.80 × 10^0^copies/µL).The minimum concentration of target DNA detected by qPCR using this dilution series as a template. Simultaneously, we compared the sensitivity of SYBR Green II real-time quantitative PCR with that of conventional PCR. This comparison provided valuable insights into the sensitivity of the real-time PCR approach and its performance in comparison to conventional PCR. Simultaneously, we employed the PCR method already established in the laboratory to confirm 20 positive clinical samples for BoAHV-1 to assess the specificity of this method. The primer sequences are listed in Table 3.

Six groups of recombinant positive plasmids were prediluted at different concentrations (ranging from 10^3^ to 10^8^ copies/µL). Under the same conditions, 3 interbatch replicates and 3 intrabatch replicates were performed. By dividing the standard deviation (SD) of each test sample by the mean value, we calculated the coefficients of variation (CV) for intrabatch and interbatch variability.

### Detection of BoAHV-1 in clinical samples

Using the SYBR Green II real-time quantitative PCR method established in this study, we performed BoAHV-1 detection on 86 nasal swab samples collected from cattle farms in Jilin Province. Simultaneously, the primers F and R were used to conduct detection using conventional PCR (Table 1) according to the reference [39], and the detection rates of the two methods were compared.

### Virus isolation

The positive nasal swab samples by qPCR were subjected to repeated freeze‒thaw cycles, followed by centrifugation, and then filtered through a 0.22-micron membrane filter for sterilization. The processed nasal swab fluid was inoculated onto MDBK cells for virus isolation. If cytopathic changes appeared during this period, the virus was passaged 5 times continuously and the TCID_50_ was tested [12]. At the same time, we utilized the laboratory-preserved BoAHV-1 viral fluid as the positive control, and supplemented DMEM with 2% horse serum as the negative control.

### Virus identification

The extracted virus samples were subjected to genome analysis using the established SYBR Green II real-time quantitative PCR assay for bovine infectious rhinotracheitis virus (BoAHV-1) detection. Additionally, viral genome samples were separately tested using conventional PCR assays for BoAHV-1, bovine viral diarrhea virus (BVDV), bovine respiratory syncytial virus (BRSV), and bovine parainfluenza virus-3 (BPIV-3) detection. To verify the results, sequencing was performed on the isolated strains.

### Phylogenetic analysis

Using BoAHV-1 JL5 strain DNA as a template, PCR amplification was performed for the gC, TK, gG, gD and gE genes. After ligation into the pMD18-T vector, the samples were sent to Shanghai Sangon Biotech Co., Ltd. for synthesis (Changchun, China) and sequencing. In this study, we utilized DNAstar software (DNAstar, Inc., located in Madison, Wisconsin, USA) to analyse and compare the nucleotide and amino acid sequences of the bacterial strain. This analysis aimed to investigate the differences between this strain and isolates from China and other countries (Table 4). We employed version 7.0 of MEGA and conducted an assessment with 1000 bootstrap replicates using the maximum likelihood method to construct the phylogenetic relationships. Ultimately, a neighbor-joining phylogenetic tree was established. Translate the JL5 nucleotide sequence into an amino acid sequence using DNAStar software. Subsequently, perform multiple sequence alignment using the Clustal W program in the Megalign module of DNAStar software.

**Table 4:** Information on the BoAHV-1 reference strains involved in the present study

Virus strain	GenBank NO	Country	separation year	Gene Alignment
NM06	JN787955.1	China	2006	TK
NM06	GU591889.1	China	2006	gG
NM06	JN787954.1	China	2006	gD
NM06 NM06	GU591891.1 JN787953.1	China China	2006 2006	gE gC
16,453/07 TN/ BoAHV-1.1	OR211605.1	Italy	2023	gC, TK, gG, gD, gE
Cooper/BoAHV-1.1	AJ004801.1	Sweden	1956	gC, TK, gG, gD, gE
VR-188/BoAHV-1.1	MF421714.1	USA	1956	gC, TK, gG, gD, gE
C43/BoAHV-1.1	MH791337.1	USA	1978	gC, TK, gG, gD, gE
C33/BoAHV-1.1	MH751901.1	USA	2013	gC, TK, gG, gD, gE
C29/BoAHV-1.1	MH751900.1	USA	1981	gC, TK, gG, gD, gE
C14/BoAHV-1.1	MH598936.1	USA	2003	gC, TK, gG, gD, gE
C18/BoAHV-1.1	MH751898.1	USA	2011	gC, TK, gG, gD, gE
NVSL challenge/BoAHV-1.1	JX898220.1	USA	-	gC, TK, gG, gD, gE
C28/BoAHV-1.1	MH598938.1	USA	-	gC, TK, gG, gD, gE
Vista IBR MLV vaccine/BoAHV-1.1	MH724206.1	USA	-	gC, TK, gG, gD, gE
TSV-2 MLV vaccine/BoAHV-1.1	MH724209.1	USA	-	gC, TK, gG, gD, gE
Express 1 MLV vaccine/BoAHV-1.1	MH724204.1	USA	-	gC, TK, gG, gD, gE
FM/BoAHV-1.1 NM14	U06934.1 MK035760.1	Spain China	- 2018	gE gE
ST/BoAHV-1.2	Z23068.1	France	1994	gD, gE
SM023/BoAHV-1.2	KM258882.1	USA	1986	gC, TK, gG, gD
SP1777/BoAHV-1.2	KM258883.1	USA	2009	gC, TK, gG, gD
B589/BoAHV-1.2	KM258881.1	USA	2001	gC, TK, gG, gD, gE
K22/BoAHV-1.2	KM258880.1	USA	1958	gC, TK, gG, gD, gE
SV507-99/BoAHV-5	NC005261.3	Brizil	1999	gC, TK, gG, gD, gE
216 II BoAHV-1.1 SHJS/BoAHV-1.2 BoAHV-1 SMU6352/BoAHV-1	KY215944.1 OP035381.1 MW805266.1 MK654724.1	India China Egypt China	1976 2020 2020 2018	gC, TK, gG, gD, gE gC, TK, gG, gD, gE gC gC

### GenBank accession numbers

The sequences obtained here of a part of the BoAHV-1 gene were submitted to the Genbank under the accession number: gD:PP277988;gG:PP277989;gC:PP277990;gE:PP277991;TK:PP277992.

### Supplementary Information

**Supplementary Material 1.**

## Data Availability

No datasets were generated or analysed during the current study

## Abbreviations

IBR: Infectious bovine rhinotracheitis
BoAHV-1: Bovine herpesvirus 1
BVDV: Bovine viral diarrhea virus
BRSV: Bovine respiratory syncytial virus
BPIV-3: Bovine parainfluenza virus type 3
BRDC: Bovine respiratory disease complex
qPCR: Quantitative PCR
CPE: Cytopathic effects
