# Supplementary material for: Establishment of a real-time fluorescent quantitative PCR detection method and phylogenetic analysis of BoAHV-1
Source: BMC Vet Res. 2024 May 7;20:180. doi: 10.1186/s12917-024-04025-8 (PMC11075196; doi:10.1186/s12917-024-04025-8)
Supplement: Supplementary file 1 — Supplementary Material 1. [file 12917_2024_4025_MOESM1_ESM.docx]

**Gels are presented in Supplementary Fig. 2 C**

**M 1 2 3 4 5 6 7 8 9 10**


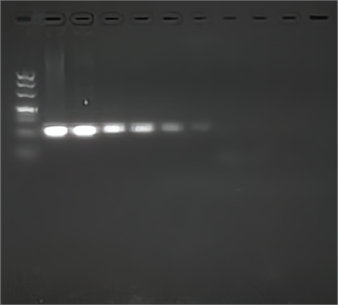


**500 bp**

**400 bp**

**300 bp**

**200 bp**

**150 bp**

**100 bp**

**50 bp**

**Sensitivity test of conventional PCR of TK; Lane M：DL500 DNA Marker; Lane M1-9:** **7.80×10^8^，7.80×10^7^，7.80×10^6^，7.80×10^5^，7.80×10^4^，7.80×10^3^，7.80×10^2^，7.80×10^1^，7.80 ×10^0^ copie/µL; 10: Negative**

**Gels are presented in Supplementary Fig. 3 B**

**
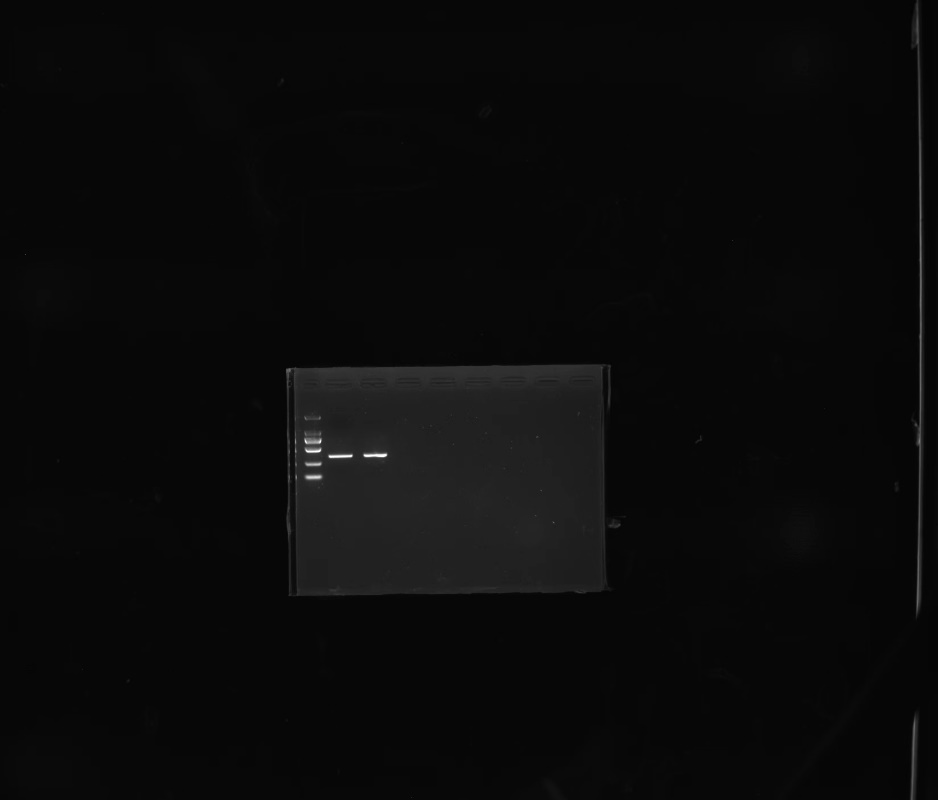
**

**M 1 2 3**  4 5 6

**2000 bp**

**1000 bp**

**750 bp**

**500 bp**

**250 bp**

**100 bp**

**Conventional PCR identification results: Lane M: DL2000 DNA marker; Lane 1: BoAHV-1 standard positive strain; Lane 2: Isolated BoAHV-1 strain; Lane 3: Negative control of BoAHV-1 (MDBK cells); Lane 4: BVDV; Lane 5: BRSV; Lane 6: BPIV-3.**
